# Clinical outcomes of surgical treatment for Copenhagen syndrome: a case series 

**DOI:** 10.1186/s13256-023-04004-x

**Published:** 2023-07-06

**Authors:** Saeid Safaei, Parisa Azimi, Taravat Yazdanian

**Affiliations:** 1 Knee and Sport Medicine Research Center, Milad Hospital, Tehran, Iran; 2 Neuroscience Research Center, Shahid Beheshti University of Medical Sciences, Tehran, Iran; 3 School of Medicine, Capital Medical University, Beijing, China

**Keywords:** Copenhagen disease, Kyphosis, Diagnosis

## Abstract

**Background:**

Copenhagen syndrome (CS) is a rare disorder mostly observed in adolescent. The onset of the disease, with a progressive anterior vertebral ankylosis in the thoracic and/or lumbar areas often clinically revealed by thoracolumbar kyphosis. We report a series of three patients of CS with good outcome.

**Case presentation:**

The mean age of patients were 14.0 (SD = 3.6) years at admission time. Patients underwent clinical and radiological examination (MRI, CT scan, and bone scan) before surgery and revealed Copenhagen syndrome. Case 2 received conservative treatment braces and regular follow-up. Finally, all patients were treated according to their clinical conditions through a combined surgical approach such as pedicle subtraction osteotomy (PSO), ponte osteotomy, hook, pedicular screw insertion, and fusion. In postoperative follow-up, the deformity correction was achieved with proper alignment in all the cases.

**Conclusion:**

The treatment of CS with PSO plus ponte osteotomy seems to result in an excellent surgical procedure and outcome for our patients based on deformity severity. Bone scan imaging could be considered as an aid to differential diagnosis, which is an effective method.

## Background

Progressive non-infectious anterior vertebral fusion (PNAVF) is a rare disorder of unknown etiology that usually presents with thoracolumbar spine ankylosis, often combined with kyphosis [1, 2]. It usually affects children and it may progress rapidly in adolescence [1]. This disease was first described in 1949 by Knutsson [3]. Since one series of 26 cases was presented in 1991 by the university hospital of Copenhagen [1], the term “Copenhagen syndrome (CS)” is used to characterize this disorder. Prevalence is unknown, but so far, about 100 patients have been reported in the literature [4]. The diagnosis of these patients is challenging [5]. Treatment may involve bracing throughout childhood to slow progression [1] or surgical correction of the deformity [2]. We describe a series of three cases. The clinical summary, imaging findings, and surgical procedures are discussed. Also, this study suggests new diagnostic and treatment approaches for these patients.

## Case reports

### Case 1

**Fig. 1:**
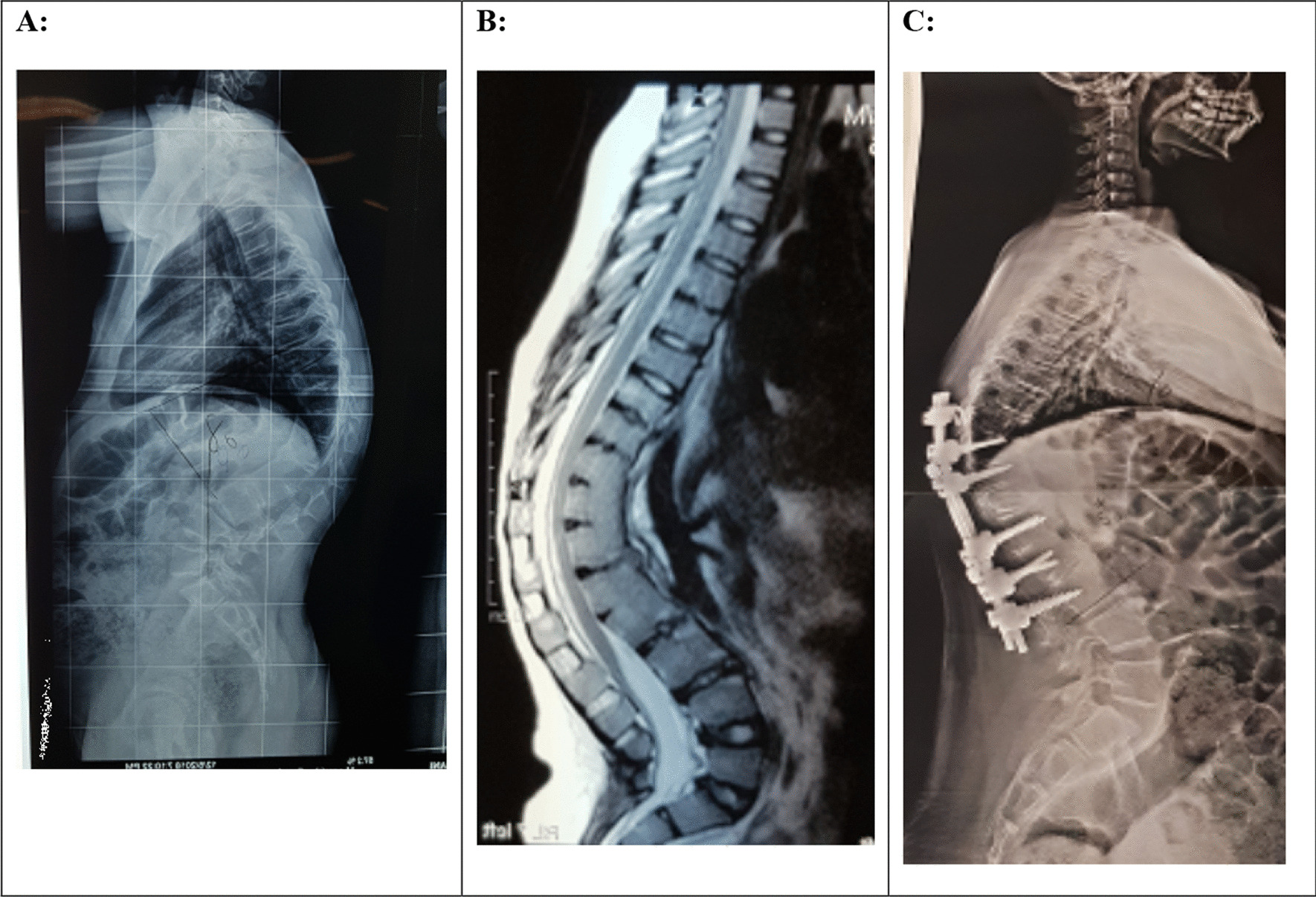
**A** preoperative lateral radiograph of the spine. There is narrowing noted at the T10/12 and L1/2 disc spaces anteriorly. **B** MR imaging with sagittal T2 sequences through the thoracolumbar spine demonstrate loss of anterior disc height and fusion at the T10/12 and L1/2 levels. **C** 6-month post-operative lateral radiograph of the spine

A Iranian female was referred at age 11 with a history of progressive thoracolumbar kyphosis and back pain. Initial examination showed a thoracolumbar kyphotic deformity and normal neurologic state. Radiography revealed kyphosis associated with some anterior disc abnormality at the T10–T12 and L1/2 levels with a kyphosis measuring 80° (Fig. 1a). Magnetic resonance imaging (MRI) study presented some focal reduction in anterior disc space and low lying cord (Fig. 1b). Laboratory tests were completely normal and the bone scan was positive. The patient underwent a T12 pedicle subtraction osteotomy (PSO), followed by a T9 hook and T10–L3 lateral screw placement with rod fixation. The deformity correction was achieved at the postoperative day 2 with a kyphosis measuring 45°. Six months postoperative radiography showed fusion of PSO site and an asymptomatic proximal junctional kyphosis (PJK) with a kyphosis measuring 64° (Fig. 1c).

### Case 2

**Fig. 2:**
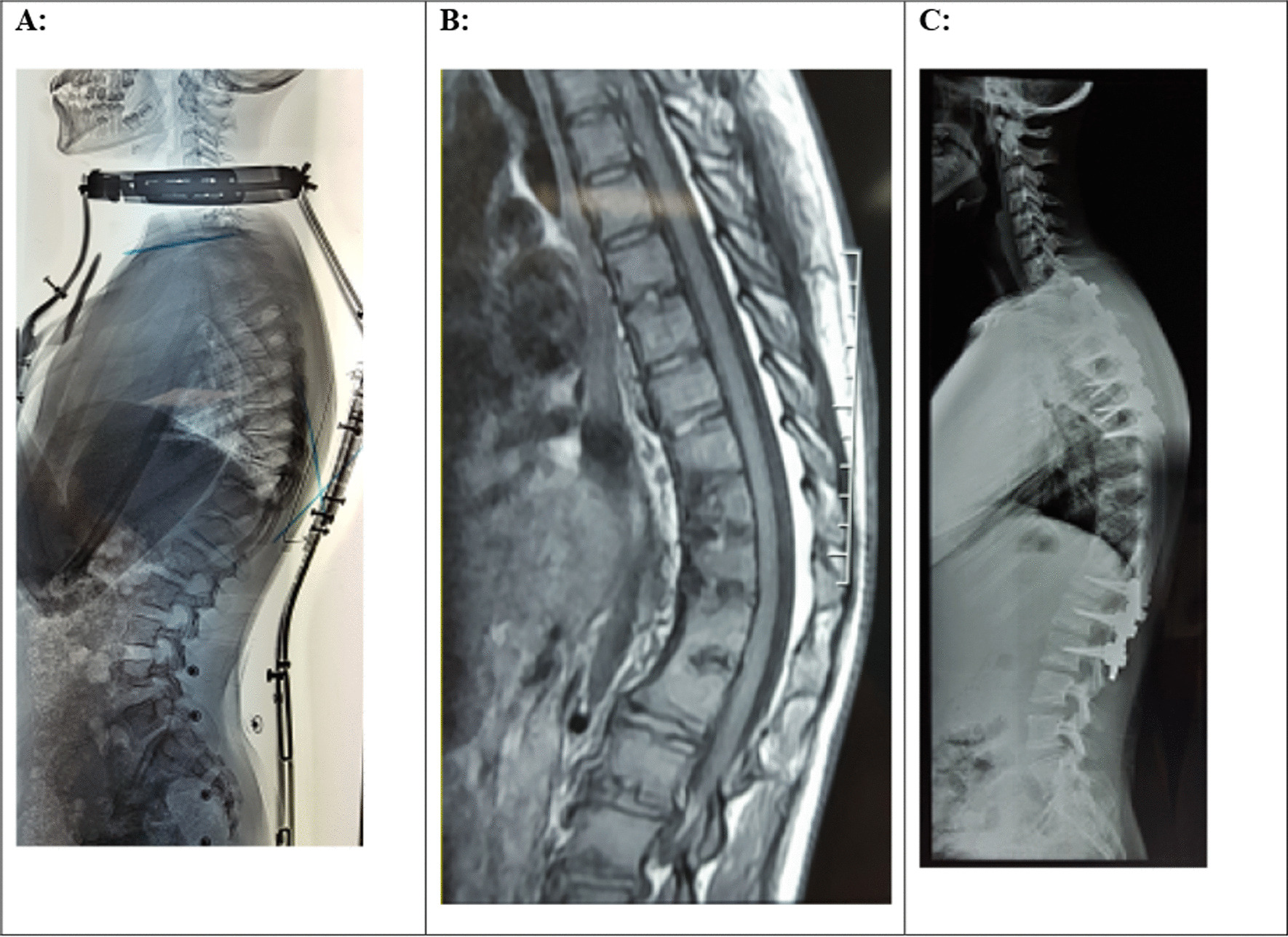
**A** preoperative lateral radiograph of the spine. There is narrowing noted at the T8/12 disc spaces anteriorly. **B** MR imaging with sagittal T1 sequences through the thoracolumbar spine demonstrate loss of anterior disc height and fusion at the T8/12 levels. **C** 5-year post-operative lateral radiograph of the spine

A Iranian male patient presented at age 12 with a T1–T12 kyphosis measuring 70°. Initial examination didn’t show any back pain and neurological deficit. Laboratory tests were normal. Due to parents’ opposition, surgery was not performed and conservative treatment was considered. On spinal brace treatment and serial clinical evaluation, the deformity persisted from the ages of 12 to 16. Subsequent radiographs and MRI showed progressive disc space narrowing and end-plate erosions anteriorly at the T8–T12 levels, and eventual anterior bony fusion by the age of 16 with a thoracic kyphosis measuring 63° (Fig. 2a, b). During brace treatment, the reduction of T1–T12 kyphosis from 70 to 63° was observed due to the effect of the brace on the upper and middle thoracic segments, which was not significant. Also, the T8–T12 kyphosis changed from 38 to 43°. A positive bone scan was observed at this age. Then, spinal surgery was performed with multilevel ponte osteotomy, T2 hook, and T3–L2 pedicular screw insertion and fusion. The deformity correction was obtained with a T1–T12 kyphosis measuring 43° at the 5-year postoperative follow-up (Fig. 2c).

### Case 3

**Fig. 3:**
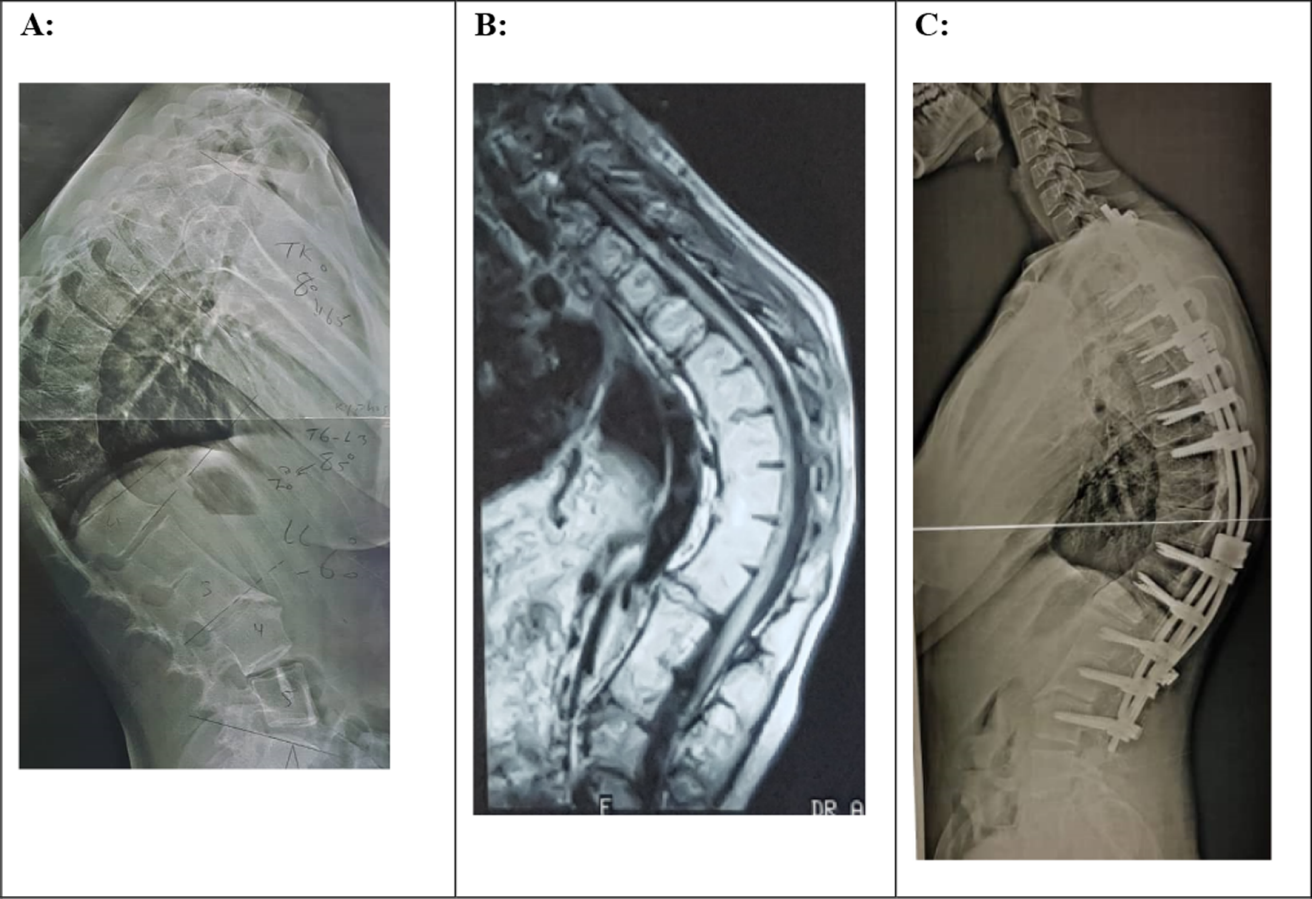
**A** preoperative lateral radiograph of the spine. There is narrowing noted at the T8/L1 and L2/3 disc spaces anteriorly. **B** MR imaging with sagittal T1 sequences through the thoracolumbar spine demonstrate loss of anterior disc height and fusion at the T8/L1 and L2/3 levels. **C** 1st day post-operative lateral radiograph of the spine

**Fig. 4:**
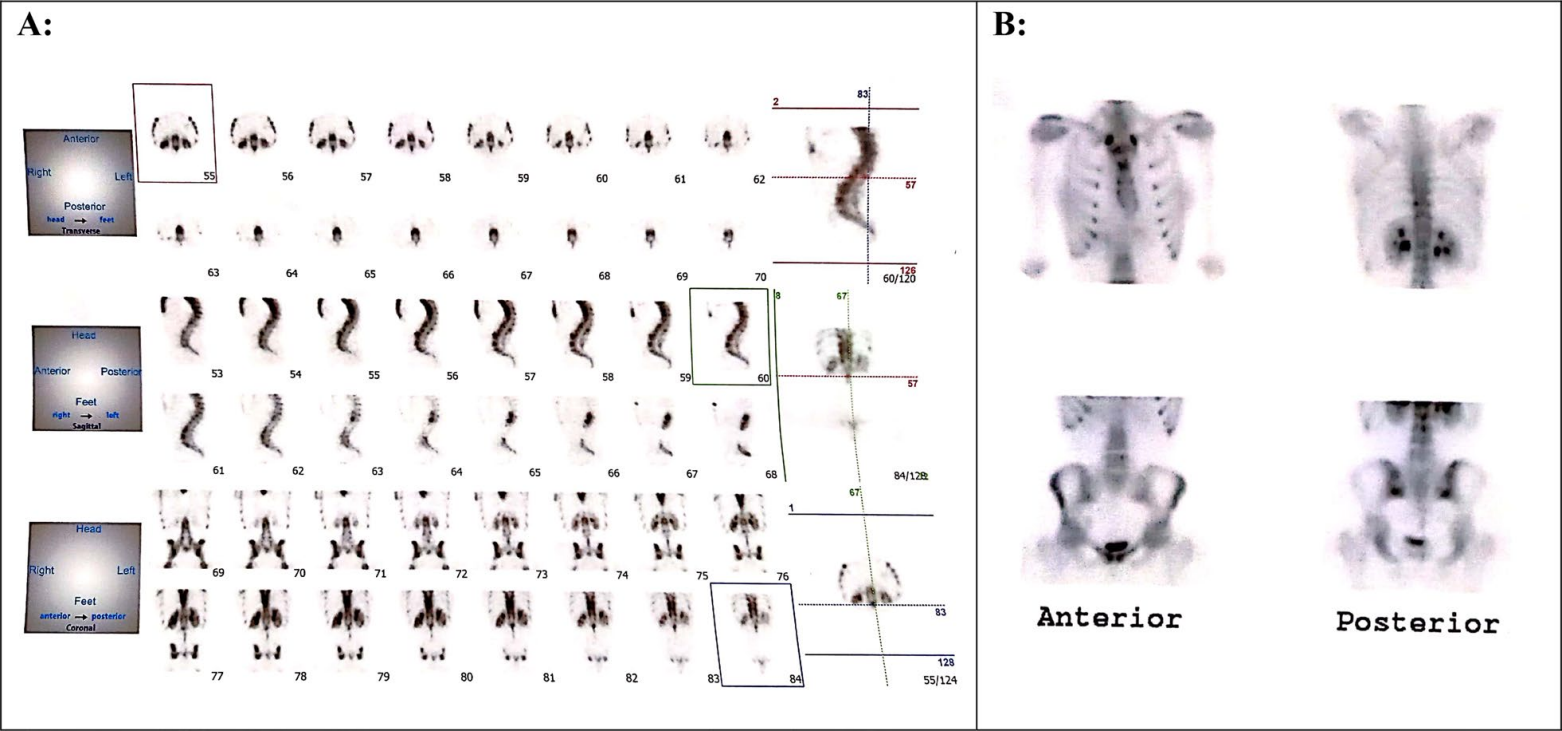
**A**, **B** distribution of the positive bone scan in case 3

A Iranian female at age 19 was presented with a history of progressive, painless thoracolumbar kyphosis after the age of 10. Other symptoms were not observed and there was no family history of spinal deformity. Her neurological status and lab tests were normal. Radiographs revealed anterior narrowing at the T8/L1 and L2/3disc spaces with a kyphosis measuring 85° (Fig. 3a). MRI showed anterior disc height loss and fusion at these levels (Fig. 3b). The patient underwent a two-stage operation. In the first stage, she had L1 modified PSO and T11–L3 pedicular screw insertion. Two days later, in the second stage, we did ponte osteotomy at T4–T8, T2 hook, and T3–L4 pedicular screw insertion and fusion. The deformity correction was obtained with a kyphosis measuring 46 (Fig. 3c), and 47° at the 1-day and 13-month postoperative follow-up, respectively. The distribution of the positive bone scan was shown in this case (Fig. 4). We performed intraoperative neuromonitoring for all our patients.

## Discussion

Copenhagen syndrome is rare in the general population; however, it is worth considering in a differential diagnosis, especially in young children with unknown etiology. Presenting symptoms of Copenhagen syndrome are often nonspecific, hence their diagnosis can be easily missed. Key findings on imaging modalities such as bone scan may be required to correctly diagnose CS patients. Besides, PSO plus ponte osteotomy may be appropriate for CS patients based on the severity of the deformity.

The accurate diagnosis of CS can be difficult. The differential diagnosis of CS is broad and includes Scheuermann’s disease, congenital kyphosis type II, anterior limbus vertebra, ankylosing spondylitis, and diffuse idiopathic skeletal hyperostosis in adults, also thalidomide embryopathy in children [5]. Clinical history, imaging studies such as thoracolumbar spine X-ray and MRI, and laboratory studies can significantly narrow this differential diagnosis [6]. MRI can detect early changes in PNAVF and it also reflects the disease activity [7], but in some cases it is controversial. Therefore, the bone scan might be a valuable tool to further differentiate these diseases.

One might inquire about the reason why the bone scan was used for the diagnosis of patients with suspected CS. Certainly, the initial idea came from the fact we used bone scan imaging in patients with ankylosing spondylitis (AS). In patients with AS, spinal fusion is started from the sacroiliac joint and lumbar spine, and the bone scan is positive due to the progression of the disease. Congenital kyphosis type II is the most important differential diagnosis of CS in clinical practice. On the other hand, serial imaging is needed for the diagnosis of patients with CS, however, some of these patients visit the clinic after the progression of the disease. Unlike CS, congenital kyphosis type II disease is a stable condition in adolescence. Also, imaging such as MRI and X-ray are often similar in congenital kyphosis type II and CS diseases. Hence, a bone scan may be useful to differentiate CS and congenital kyphosis type II disease. Further studies are needed to confirm this finding.

Then, we used thoracolumbar spine X-ray, MRI, and bone scan imaging combined with clinical history results to diagnose disease. In this study, we used a bone scan of the spine and has proven to be a clinically useful part of the investigation of patients with CS. Furthermore, a bone scan is useful in the evaluation of back pain where other imaging modalities are negative, does not correlate with the clinical outcomes, or has too many abnormalities. Increasing experience with bone scan imaging in CS patients may allow more accurate disease monitoring. There exists a potential for future evaluation with other nuclear medicine imaging modalities, to establish whether bone scan can provide prognostic information.

Surgery is the treatment of choice for patients with CS disease, although preoperative treatment with bracing throughout childhood may slow the progression of deformity [1, 2]. Researchers have also stated the option of treatment method based on five stages of disease progression in CS [4, 8], which is in line with our findings. To monitor treatment progression, individuals should be closely observed. Surgical procedure should only be reserved for those cases in whom severe deformity and/or progression of disease is confirmed [9]. In patients with CS, surgery was performed with a combination of anterior and posterior approach [4], or posterior approach only [10].

The main indication for PSO is large sagittal deformities especially when they are rigid [11]. PJK is a complication of surgical management of spinal deformity with a multifactorial etiology. Also reducing the osteotomy angle and extending fusion to higher levels can decrease the risk of PJK [12]. We used ponte osteotomy in two cases. Using the ponte technique for upper unfused levels after PSO can be helpful to correct the deformity, and to avoid the complications of anterior release surgery. In this study, PSO plus ponte osteotomy was applied in case 3, with most correction of deformity compared to other cases. Based on our experience, PSO is applied in the apex of the deformity for more correction, but using PSO only may increase the risk for PJK. Using ponte for upper unfused levels after PSO, improve spinal alignment and decrees the risk for PJK. Hence, combination of PSO and ponte osteotomy can result in is correct of sagittal balance compared with PSO alone, especially, in patients with severe thoracolumbar kyphosis.

## Conclusions

Our experience suggests that PSO plus ponte osteotomy is an effective treatment that can correct deformity based on the severity of the deformity. In addition, bone scan imaging is shown to reflect well the progression of CS. Further studies are needed to evaluate the efficacy of bone scan diagnosis in these patients.

## Data Availability

All the data supporting our findings are contained within the manuscript.

## Abbreviations

CS: Copenhagen syndrome
PNAVF: Progressive non-infectious anterior vertebral fusion
PSO: Pedicle subtraction osteotomy
PJK: Proximal junctional kyphosis
